# Efficacy and safety of prophylactic use of ketamine for prevention of postanesthetic shivering: a systematic review and meta analysis

**DOI:** 10.1186/s12871-019-0910-8

**Published:** 2019-12-30

**Authors:** Yang Zhou, Abdul Mannan, Yuan Han, He Liu, Hui-Lian Guan, Xing Gao, Ming-Sheng Dai, Jun-Li Cao

**Affiliations:** 1 Department of Anesthesiology, The Affiliated Hospital of Xuzhou Medical University, Xuzhou, 2210002 Jiangsu China; 2 Jiangsu Province Key Laboratory of Anesthesiology, Xuzhou Medical University, Xuzhou, 221004 Jiangsu China

**Keywords:** Postanesthetic shivering, Anti-shivering, Ketamine

## Abstract

**Background:**

Postanesthetic shivering is a common complication of anesthesia, which accounts for much discomfort in postoperative patients and may increase postoperative complications in high-risk patients. Due to the lack of high-quality evidence, it is difficult to draw a conclusion about optimal anti-shivering medication. The main purpose of this meta-analysis was to analyze and evaluate the efficacy and safety of prophylactic use of ketamine for preventing postanesthetic shivering.

**Methods:**

We searched the following databases: Medline, Embase, and the Cochrane Central Register of Controlled Trails for randomized controlled trials. The primary outcome observed was the difference of the incidence rate of postanesthetic shivering between ketamine group and placebo group. The secondary outcomes were the sedation score and incidence of the side effects caused by ketamine and any other drugs utilized in the studies.

**Results:**

In this meta-analysis, we analyzed a total of 16 trials including 1485 patients. Ketamine reduced the incidence rate of postanesthetic shivering compared to a placebo (odds ratio [OR]: 0.13, 95% confidence interval [CI]: 0.06 to 0.26, P<0.01). Regarding side effects, there was no evident variability of the incidence of nausea and vomiting. Usage of ketamine was associated with a lower rate of hypotension and bradycardia when compared to a placebo. Hallucinations were more frequently observed in patients who received higher doses of ketamine. No significant difference was found in the incidence of postanesthetic shivering with ketamine versus other pharmacological interventions.

**Conclusions:**

Ketamine can prevent postanesthetic shivering without severe side effects. However, ketamine shows no advantage over other anti-shivering drugs.

## Background

Postanesthetic shivering is a frequent complication of anesthesia, perhaps even aggravating pain. It is characterized by involuntary movement that may affect one or more muscle groups and is a very unpleasant and physiologically stressful experience. Postanesthetic shivering can interfere with electrocardiography (ECG) and oxygen saturation (SpO2) monitoring [1]. More importantly, it can increase oxygen consumption combined with minute ventilation and carbon dioxide production. Moreover, it is believed that postanesthetic shivering can increase mortality in the elderly and patients with coronary artery disease [2].

The aetiology of shivering is not sufficiently understood. Thermoregulatory and non-thermoregulatory factors may contribute to postoperative shivering including exposure to cold weather, inadequate pain control, and opioid withdrawal [3, 4]. The gold standard for treatment and prevention has not yet been defined. A variety of pharmacological treatments and methods to reduce postoperative shivering have been used including meperidine, alfentanil, tramadol, magnesium sulfate, ondansetron, dolasetron, and dexmedetomidine [5–9]. Ketamine has also been used as an anti-shivering drug. It is a non-competitive N-methy-D-aspartate (NMDA) receptor antagonist; it may prevent postanesthetic shivering by decreasing core-to-peripheral heat distribution. Although many published literatures have investigated the potential effects of ketamine for prevention of postanesthetic shivering, there is no consensus regarding the appropriateness of this drug. Thus, an evidence-based understanding of the benefits and risks of ketamine would identify its rational and optimal use. We conducted the meta-analysis to assess the efficacy and safety of ketamine for the prevention of shivering in patients undergoing various surgical procedures.

## Methods

This meta-analysis was conducted and reported according to the Preferred Reporting Items for Systematic Reviews and Meta-Analysis (PRISMA) guidelines.

### Search strategy

Two authors (Y.Z., A.M.) independently searched MEDLINE (2000 to March 2018), EMBASE (2000–2018), and the Cochrane Central Register of Controlled Trails (March 2018) with no language restrictions. By reviewing the references of the eligible articles, we identified additional studies relevant to our meta-analysis. The following search-term strategy was used:

1) shivering; 2) tremor; 3) shake; 4) hypothermia; 5) anesthesia; 6) postanesthetic; 7) postoperative; 8) surgery; 9) ketamine; 10) 1 or 2 or 3 or 4; 11) 5 or 6 or 7 or 8; 12) 9 and 10 and 11.

### Criteria for considering studies for this review

The selection criteria were pre-established. Inclusion criteria were: (1) controlled clinical trial; (2) prophylactic use of ketamine compared with a placebo or other pharmacological interventions; (3) reported the incidence of postoperative shivering. Trials were not considered for the following reasons: (1) other anti-shivering drugs were also administrated during the anesthetic induction or maintenance period besides ketamine; (2) data from abstracts, letters, or reviews. We included any participants undergoing operative procedures with general or spinal anesthesia. The following outcomes were measured: (1) incidence of postanesthetic shivering; (2) sedation score; (3) incidence of other side effects.

### Data collection and analysis

Two review authors (Y.Z., A.M.) independently screened all the titles and abstracts of the studies during the initial search to identify the included studies. After removing the duplicates, potentially relevant studies were retrieved in full-text version for the further assessment. We resolved any disagreement by discussion with another author (G. H. L) of our group.

Data extraction was conducted by two authors (Y.Z., A.M.) independently using the data collection form established previously. The following data were collected from each study: primary author, publication year, anesthetic methods, demographic characteristics of participants, surgery types, comparisons, and other non-pharmacological warming methods. We recorded the number of patients experiencing shivering in each group for dichotomous data.

We used the Review Manager software of the Cochrane Collaboration (RevMan 5.2) to perform the quantitative analysis. The results of dichotomous data are expressed as odds ratio (OR) and 95% confidence intervals (CIs). Heterogeneity testing was performed with Z score and X^2^ statistical analysis; *P* < 0.1 was considered to indicate heterogeneity. The fixed effect model or the random effect model were applied according to the heterogeneity of the study. A fixed effect model was used when I^2^ < 50%. We reported the results of included studies when the pooled analysis was not appropriate. Sensitivity and subgroup analysis were performed to explore the reason for the heterogeneity. Subgroup analysis was conducted based on the anesthetic methods, various doses of ketamine used, and the types of surgery. Publication bias was evaluated by Begg’s test using Stata 13.1 software (Stata, College Station, TX, USA).

## Results

### Search results and characteristics of the studies

**Fig. 1:**
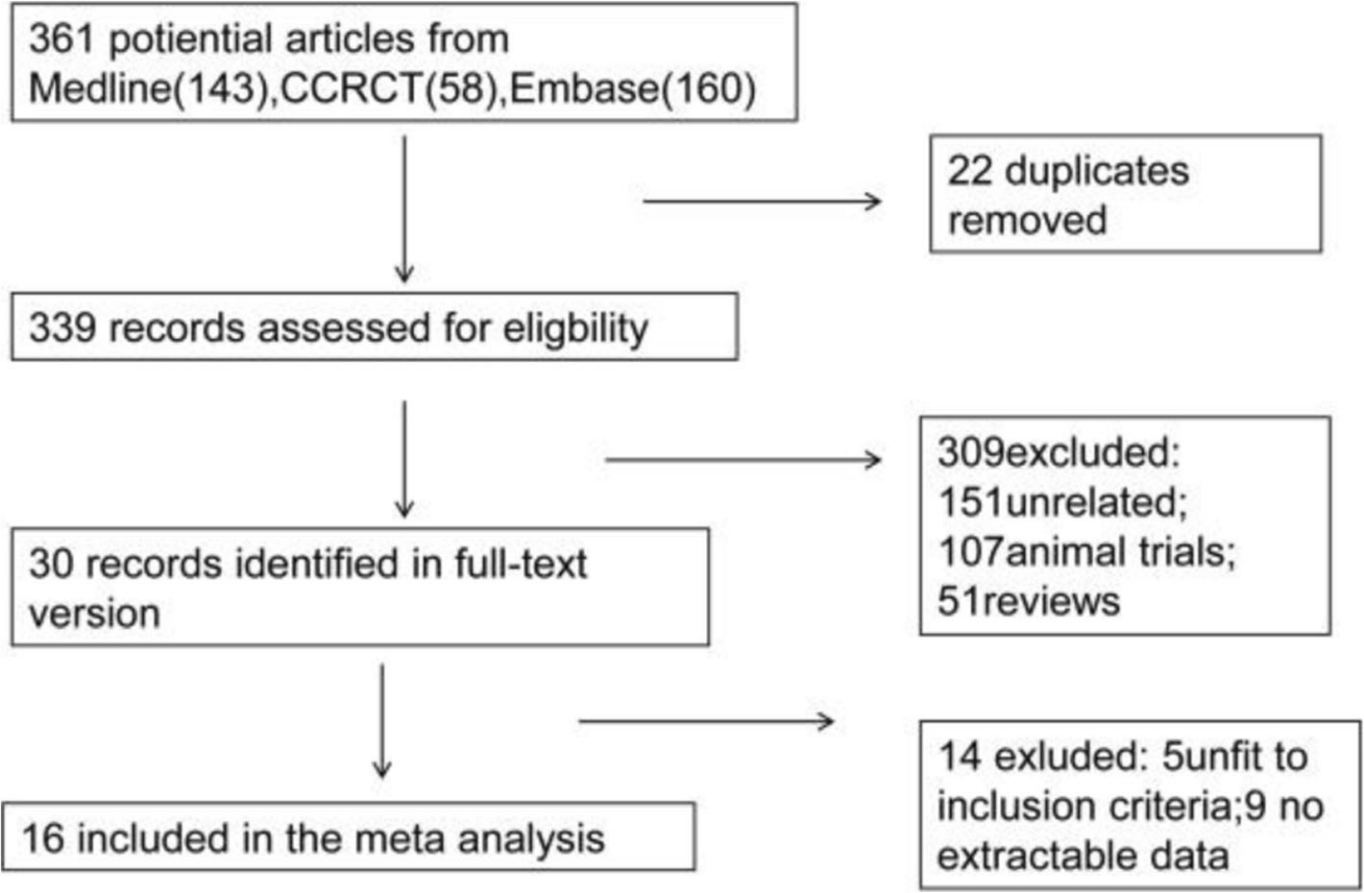
Flow diagram showing the process of studies selection

The flow chart (Fig. 1) shows the process by which the selected studies were searched. A total of 361 potential articles were identified. We reviewed 30 full-text articles, after screening the titles and the abstracts. A total of 16 [10–25] studies including 1485 patients met our selection criteria and were included in the analysis (Table 1).

**Table 1:** Summary of Characteristics of Included Studies

Study ID	Participants	Surgery Types	Anesthesia Methods	Comparison		Non-pharmacological Warming Methods
				Control Group	Intervention Group	
Sagir 2007	160 patients 18-65 yr	urological surgery	SA	C(40): saline iv	K(40):ketamine 0.5 mg/kg iv;G(40):granisetron 3 mg,iv;KG(40) ketamine 0.25/kg + granisetron 1.5 mg iv	All patients were covered with drapes and a cotton blanket. Fluids were preheated to 37^o^c
Honarmand 2008	120 patients 18-60 yr	orthopaedic surgery	SA	C(30) saline iv; K(30) ketamine 0.5 mg/kg iv;	M(30) midazolam 75 μg/kg iv; KM(30) ketamine 0. 25 mg/kg + midazolam 37.5 μg/kg iv	Fluids were preheated to 37^o^c
Zahra 2008	120 patients 5-12 yr	tonsillectomy surgery	GA	C(40):saline	K(40):ketamine 1 mg/kg;P(40):pethidine 0.5 mg/kg	None.
Han 2010	93 patients 51-78 yr	transurethral resection of the prostate	SA	C(31): epidural 0.75% ropivacaine	K1(32): epdural ketamine 0.2 mg/kg + 0.75%ropivacaine; K2(30): epidural ketamine 0.4 mg/kg + 0.75%ropivacaine	None
Shakya 2010	120 patients	Lower abdominal surgery	SA	C(40):saline iv	K(40): ketamine 0.25 mg/kg iv;O(40):ondansetron 4 mg iv	Patients were covered with standard single blanket
Ayatollahi 2011	120 patients 20-50 yr	endoscopic sinus surgery	GA	C(30): saline iv	K1(30): 0.3 mg/kg iv; K2(30): 0.5 mg/kg iv; M(30): meperidine 0.4 mg/kg iv	Patients were covered with a cotton blanket
Norouzi 2011	120 patients 18-65 yr	elective orthopedic surgery	GA	C(30):saline iv	K1(30):ketamine 0.125 mg/kg iv;K2(30):ketamine 0.25 mg/kg iv;K3(30):ketamine 0.5 mg/kg iv	None
Wason 2012	200 patients 18-65 yr	ower abdominal or lower limb surgery	SA	C(50):saline iv	K(50):ketamine 0.5 mg/kg iv;C(50):clonidine 75mcg;T(50):tramadol 0.5 mg/kg iv	Fluids were preheated to 37^o^c
Zavareh 2012	135 patients 18–70 yr	elective surgery	GA	K(45):ketamine 0.5 mg/kg iv;	P(45):pethidine 0.5 mg/kg;D(45):dexamethasone,0.6 mg/kg	None
Abdelhalim 2014	120 patients 18-45 yr	ENT surgery	GA	C(30): saline iv	O(30): ondansetron 8 mg iv; K(30): ketamine 0.5 mg/kg iv; OK(30) ondansetron 8 mg + ketamine 0.25 mg/kg iv	None
Petskul 2016	183 patients 18-65 yr	orthopedic surgery	GA	C(92):saline iv	K(91):ketamine 0.25 mg/kg iv	All patients were warmed by air force warmer
Mohtadi 2016	117 patients 18-40 yr	cesarean section	SA	C(39):saline iv	K(39):ketamine 0.25 mg/kg,iv;O(39):ondansetron 4 mg,iv	None
Hasannasab 2016	120 patients 20-45 yr	gynecologic surgery	GA	K(40): ketamine 0.25 mg/kg iv;	M(40): meperidine 20 mg iv; D(40): doxapram 0.25 mg/kg iv	Patients were covered with a standard blanket
Lakhe 2017	120 patients 18–65 years	gynecological and orthopedic surgery	SA	C(30):saline,iv	T(30):tramadol 0.5 mg/kg iv O(30):ondansetron 4 mg,iv;K(30):ketamine 0.25 mg/kg iv	Patients were covered with drapes
Lema 2017	123 patients 18-39 yr	cesarean section	SA	C(41):saline iv	K(41):ketamine 0.2 mg/kg iv;T(41): tramadol 0.5 mg/kg iv	Patients were covered with drapes

Abbreviations: *yr* years; *GA* general anesthesia; *SA* spinal anesthesia; *C* control;*O* ondansetron; *T* tramadol; *M* meperidine; *D* doxapram; *G* granisetron; *CL* clonidine; *P* pethidine

In 15 trials, participants were adults. One trial [23] included children aged 5–12 years. Participants in seven trials [10, 12, 13, 18, 19, 23, 24] underwent operations under general anesthesia; participants in nine trials [11, 14–17, 20–22, 25] were under spinal anesthesia. In 13 trials [10, 12, 14–23] ketamine was compared to placebo; in 4 trials [12, 13, 23, 24] ketamine was compared to pethidine; in the other 4 trials [11, 15, 16, 22] ketamine was compared to tramadol. Ketamine was also compared to ondansetron in 4 trials [10, 15, 17, 21].

The administration time and routes were different among included trials. In 10 trials [11, 14–17, 20–23, 25] the intervention drugs were given immediately after induction of anesthesia or intrathecal injection; in five trials [10, 12, 18, 19, 24] drugs were administrated before completion of the surgical procedure; in one trial [13] patients received study drugs before wound closure. Study drugs were given as an IV bolus in 14 trials [10–22, 24]; in two trials, patients received the study drugs epidurally [25] or intramuscularly [24]. In four trials [14, 15, 18, 19], patients underwent orthopedic surgery; patients in two studies [21, 22] underwent abdominal surgery; patients in two studies [16, 17] underwent cesarean section; patients in two studies [20, 25] underwent urological surgery; in three trials, participants received ENT surgery [10], endoscopic sinus surgery [12], or tonsillectomy surgery [23].

Regarding measurement of intensity of shivering in a patient, 13 trials [10, 13–17, 19–25] utilized a scale with variation points ranging from 0 to 4: 0 = no shivering; 1 = piloerection or peripheral vasoconstriction, but no visible shivering; 2 = muscular activity in only one muscle group; 3 = muscular activity in more than one muscle group, but not generalized; 4 = shivering involving the whole body. One trial [12] applied a 0–3 scale for evaluating the intensity: 0 = no shivering; 1 = mild fasciculation of face or neck muscles; 2 = visible tremor involving more than one muscle; 3 = gross muscular activity involving the entire body. Two studies [11, 18] did not assess the intensity of postanesthetic shivering.

### Assessment of the risk of bias in the included studies

Two authors (Y.Z., A.M.) independently assessed the following domains using the Cochrane ‘Risk of bias’ tool:

- Sequence generation
- Allocation concealment
- Blinding of participants, personnel
- Blinding of outcome assessment
- Incomplete outcome data
- Selective outcome reporting
- Other bias

**Fig. 2:**
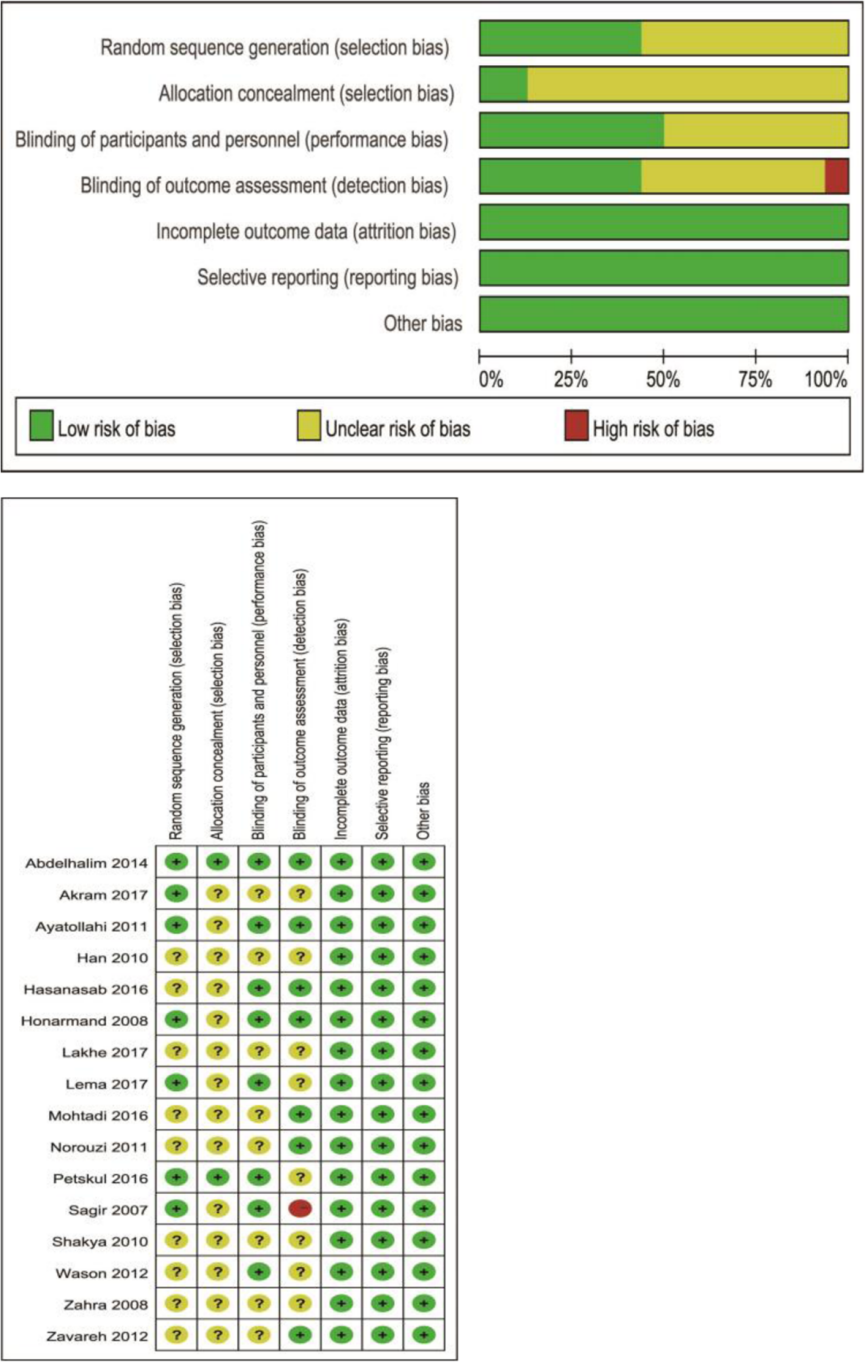
Risk of bias graph and summary

We completed ‘Risk of bias’ figures for each included study (Fig. 2). See more details in Appendix.

### Publication bias

Begg’s test showed that there was no publication bias for the primary outcome (*p* = 0.055).

#### Effects of interventions

##### Primary outcome

**Ketamine vs placebo**

**Fig. 3:**
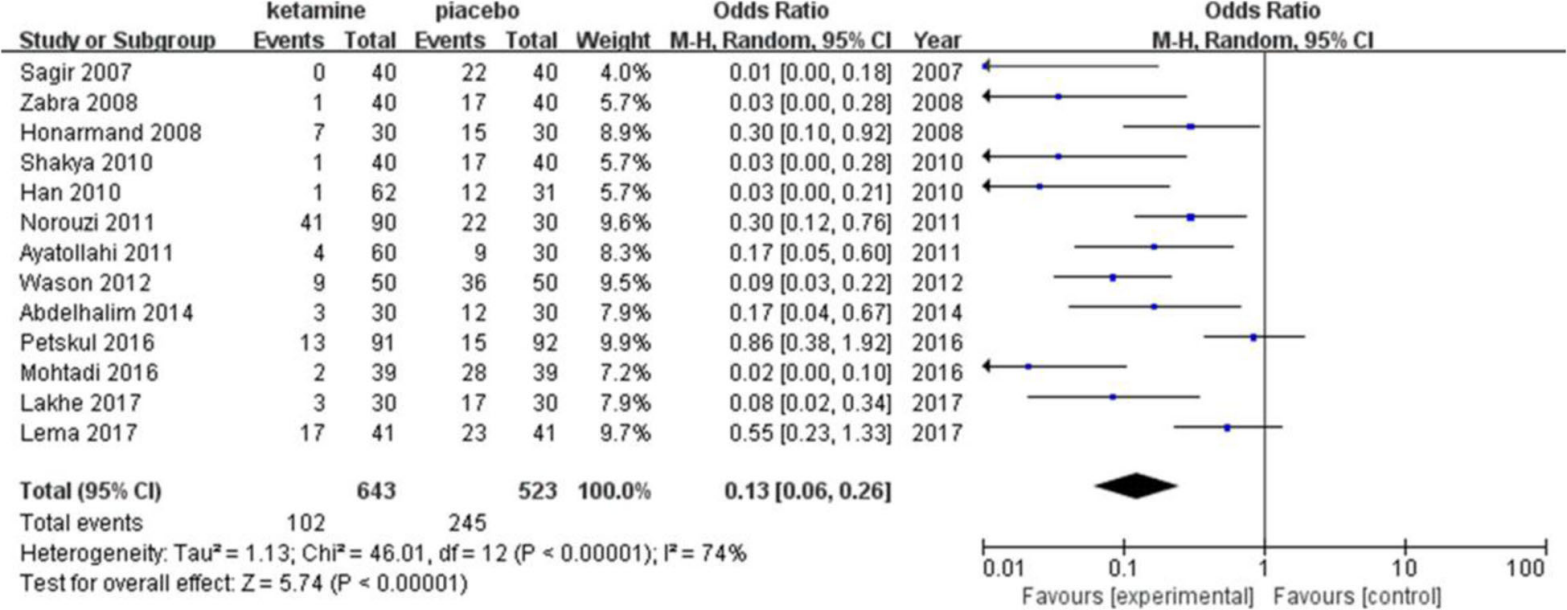
Forest plots of effects of ketamine on postanesthesia shivering. CI indicates confidence interval

**Fig. 4:**
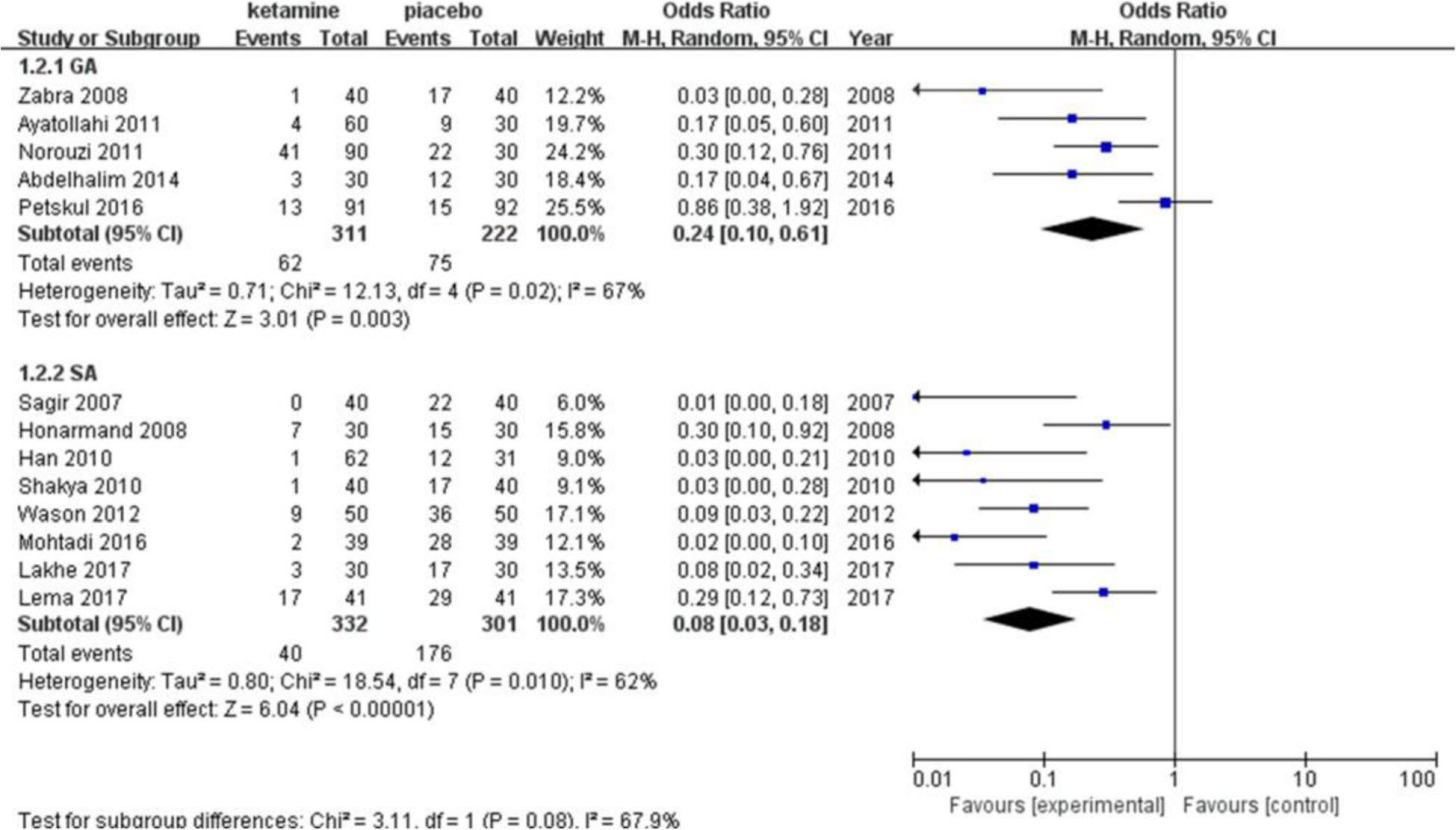
Result of subgruop analysis according to different anesthetic methods. CI, confidence interval; GA, genaral anesthesia; SA, spinal anesthesia

**Fig. 5:**
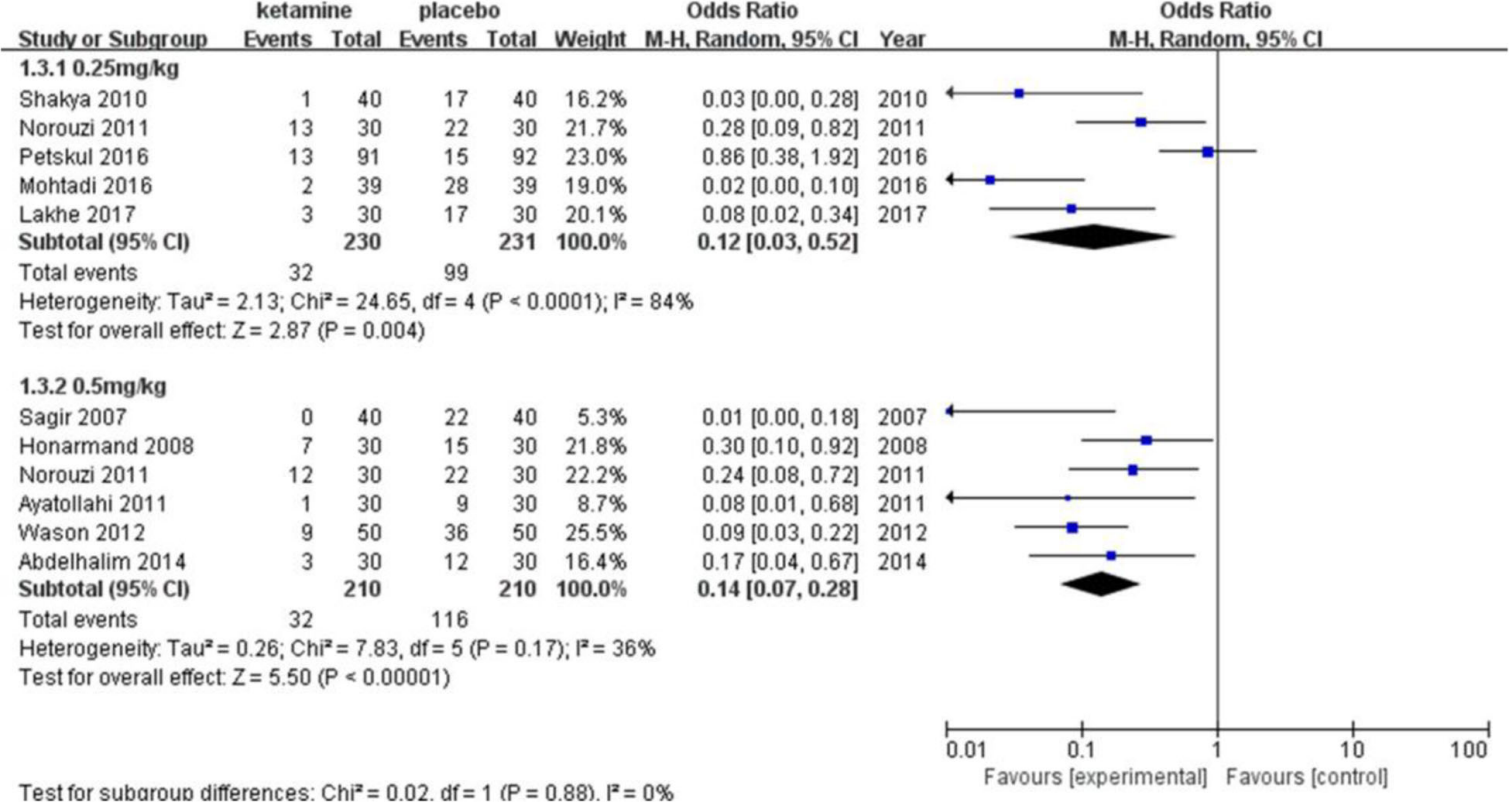
Result of subgruop analysis according to different doses of ketamine administrated. CI indicates confidence interval

**Fig. 6:**
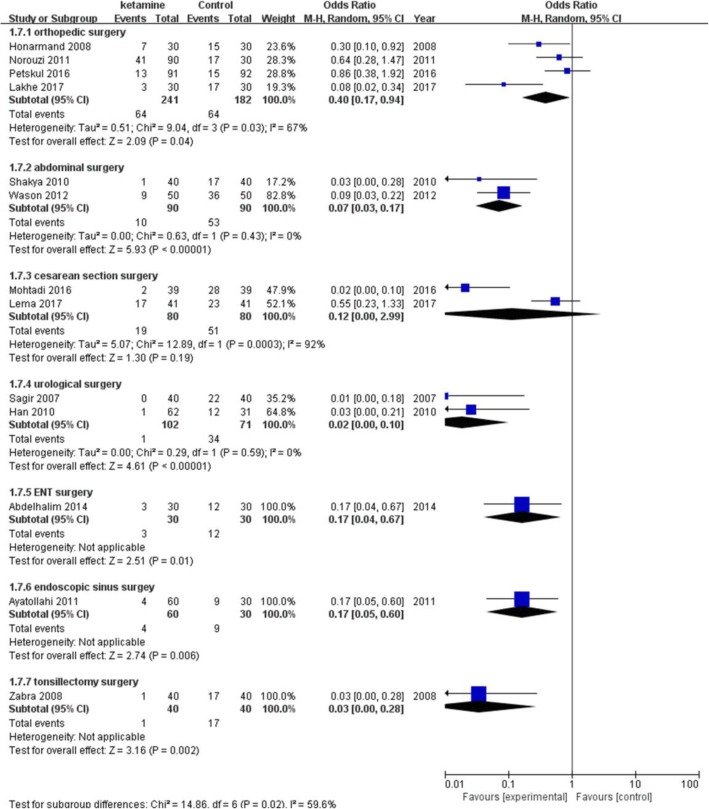
Result of subgruop analysis according to different types of surgries. CI indicates confidence interval

The incidence of postanesthetic shivering was compared between ketamine and a placebo in 13 trials including 1166 patients (Fig. 3). Ketamine has been shown to significantly decrease the incidence of shivering (pooled OR = 0.13; 95% CI: 0.06 to 0.26, *P* < 0.00001). There was significant and prominent heterogeneity for this outcome (I^2^ = 74%). Begg’s test showed that there was no risk of publication bias (*P* = 0.06). A subgroup analysis was performed to explore the evidence-based reason. In subgroup analysis of anesthetic methods, the heterogeneity was 67% in the GA (general anesthesia) group (Fig. 4). Sensitivity analysis was performed; a trial [22] was removed which utilized an air forced warmer intraoperatively, which showed a similar result favoring ketamine (pooled OR = 0.18; 95% CI: 0.09 to 0.37) and decreased heterogeneity (I^2^ from 67 to 21%). Ketamine reduced the incidence of postanesthetic shivering in general anesthesia (pooled OR = 0.13; 95% CI: 0.06 to 0.26) and in spinal anesthesia (pooled OR = 0.08; 95% CI: 0.03 to 0.18). (Fig. 5) shows the subgroup analysis based on the dose of ketamine used in the included trials. Ketamine reduced the incidence of postanesthetic shivering at the dose of 0.25 mg/kg (pooled OR = 0.12; 95% CI: 0.03 to 0.52) and at the dose of 0.5 mg/kg (pooled OR = 0. 14; 95% CI: 0.07 to 0.28). We performed a subgroup analysis based on the type of surgery, as this may influence the incidence of postanesthetic shivering (Fig. 6). Ketamine significantly lowered the incidence of postanesthetic shivering in patients after orthopedic surgery (pooled OR = 0.32; 95% CI: 0.13 to 0.77). Among patients undergoing abdominal, cesarean section, urological, ENT or endoscopic surgery, ketamine also reduced the incidence of postanesthetic shivering.

**Ketamine vs other pharmacological interventions**

**Fig. 7:**
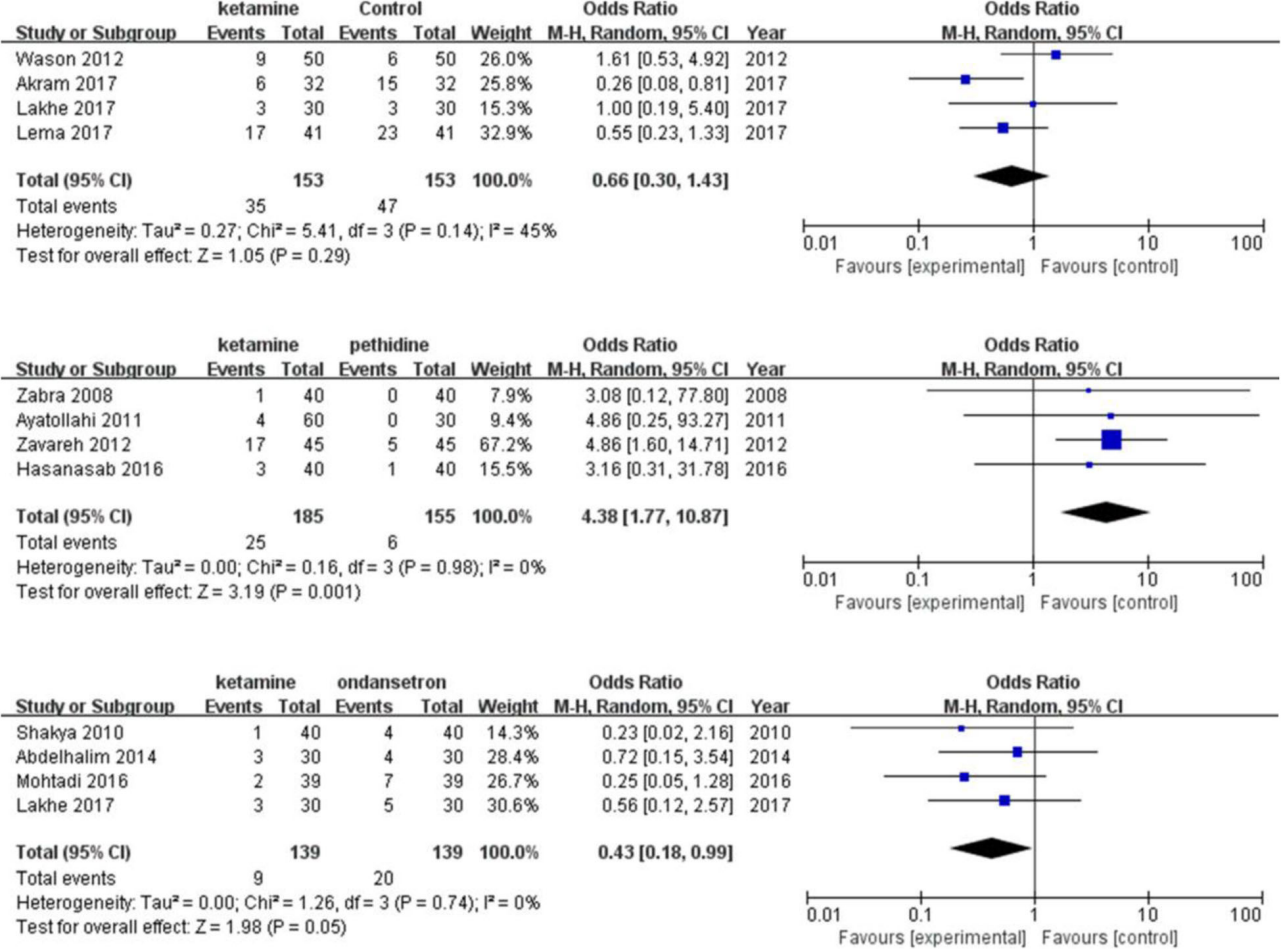
Forest plots of effects of ketamine on postanesthesia shivering compared with other study drugs. CI indicates confidence interval

A total of four studies [12, 13, 23, 24] investigated the effect of ketamine on the prevention of shivering compared with pethidine. The pooled analysis showed a definite difference in favor of pethidine (pooled OR = 4.38; 95% CI: 1.76 to 10.92). No significant difference in postanesthetic shivering was found between ketamine and other pharmacological interventions (Fig. 7).

#### Secondary outcomes

**Ketamine vs placebo**

Except for the other side effects (hypotension, bradycardia, hallucination), there was no significant difference in the incidence of postoperative nausea and vomiting (PONV) between ketamine and the placebo (pooled RR = 0.72; 95% CI: 0.48 to 1.08) (Table 2). Ketamine reduced the incidence of hypotension and bradycardia compared with the placebo (pooled RR = 0.28; 95% CI: 0.17 to 0.47; pooled RR = 0.18; 95% CI: 0.05 to 0.65). The incidence of hallucination was more significant and prevalent in the patients who received 0.5 mg/kg ketamine (Table 3); there was only one episode of hallucination in patients receiving 0.25 mg/kg. A pooled analysis was not performed because of the lack of uniform sedation score scales in the trials. All of these studies showed that the patients in the ketamine group were more sedated compared to the placebo group. Five trials [14, 16, 17, 19, 23] reported a significant decrease in core temperature in both the ketamine and placebo groups compared to the baseline temperature of participants. However, it was not significant between groups, at any time point. Three trials [20–22] reported a significant difference in core temperature between ketamine and the placebo; a greater decrease in temperature was found in the placebo group.

**Table 2:** Comparisons of incidence of other side effects

Side effccts	Number of studies	Ketamine	Placebo	
		Events/Total	Events/Total	Odds Ratio (95% CI)
Nausea and vomitting	11	48/523	57/463	0.70 [0.44, 1.12]
Hypotension	6	23/302	61/271	0.30 [0.18, 0.49]
Bradycardia	2	3/112	11/81	0.14 [0.04, 0.52]
Hallucination	5	16/272	0/151	4.41 [1.14, 17.07]

Abbreviations: *CI* confidence interval

**Table 3:** Episodes of hallucination based on the dose of ketamine

Study ID	Dose of ketamine	Ketamine	Placebo
		Events/Total	Events/Total
Honarmand 2008	0.5 mg/kg	3/30	0/30
Han 2010	0.4 mg/kg	2/30	0/31
Norouzi 2011	0.25 mg/kg	1/30	0/30
Norouzi 2011	0.5 mg/kg	4/30	0/30
Ayatollahi 2011	0.5 mg/kg	3/30	0/30
Abdelhalim 2014	0.5 mg/kg	1/30	0/30

**Ketamine vs other pharmacological interventions**

No significant difference was found in the incidence of PONV between ketamine and pethidine (pooled OR = 0.88; 95% CI: 0.38 to 2.07). Compared to tramadol, the difference in the incidence of PONV and hypotension is not significant (OR = 0.57; 95% CI: 0.18 to 178; OR = 0.90; 95% CI: 0.36 to 2.24). The incidence of PONV was higher in the ketamine group than the ondansetron group (OR = 4.49; 95% CI: 1.24 to 16.21). However, ketamine showed a lower incidence of hypotension compared to ondansetron (OR = 0.09; 95% CI: 0.00 to 3.23). Core temperature changes were reported graphically; there was no significant difference between ketamine and other pharmacological interventions.

**Summary of findings and quality of evidence**

The Summary of findings with GRADE recommendations are shown in Table 4.

**Table 4:** Summary of findings with GRADE recommendations

ketamine for postoperative shivering						
Patient or population: patients with postoperative shivering						
Settings: hospitals						
Intervention: ketamine						
Outcomes	Illustrative comparative risks^a^ (95% CI)		Relative effect (95% CI)	No of Participants (studies)	Quality of the evidence (GRADE)	Comments
	Assumed risk	Corresponding risk				
	Control	Ketamine				
Incidence of shivering	Study population		OR 0.13 (0.06 to 0.26)	1166 (13 studies)	⊕ ⊕ ⊝⊝ low^1^	
	468 per 1000	103 per 1000 (50 to 186)				
	Moderate					
	500 per 1000	115 per 1000 (57 to 206)				
Nausea and vomitting	Study population		OR 0.7 (0.44 to 1.12)	986 (11 studies)	⊕ ⊕ ⊝⊝ low^1^	
	123 per 1000	89 per 1000 (58 to 136)				
	Moderate					
	125 per 1000	91 per 1000 (59 to 138)				
Hypotension	Study population		OR 0.3 (0.18 to 0.49)	573 (7 studies)	⊕⊝⊝⊝ very low^1^	
	225 per 1000	80 per 1000 (50 to 125)				
	Moderate					
	200 per 1000	70 per 1000 (43 to 109)				
Bradycardia	Study population		OR 0.14 (0.04 to 0.52)	193 (2 studies)	⊕⊝⊝⊝ very low^1^	
	136 per 1000	22 per 1000 (6 to 76)				
	Moderate					
	165 per 1000	27 per 1000 (8 to 93)				
Hallucination	Study population		OR 4.41 (1.14 to 17.07)	423 (5 studies)	⊕⊝⊝⊝ very low^1^	
	0 per 1000	0 per 1000 (0 to 0)				
	Moderate					
	0 per 1000	0 per 1000 (0 to 0)				

^a^The basis for the assumed risk (e.g. the median control group risk across studies) is provided in footnotes. The corresponding risk (and its 95% confidence interval) is based on the assumed risk in the comparison group and the relative effect of the intervention (and its 95% CI)

*CI* Confidence interval; *OR* Odds ratio;

GRADE Working Group grades of evidence

High quality: Further research is very unlikely to change our confidence in the estimate of effect

Moderate quality: Further research is likely to have an important impact on our confidence in the estimate of effect and may change the estimate

Low quality: Further research is very likely to have an important impact on our confidence in the estimate of effect and is likely to change the estimate

Very low quality: We are very uncertain about the estimate

## Discussion

In the present study, we compared different randomized controlled trials to identify the beneficial aspects of ketamine. We compared the usage of ketamine and its relevance in anaesthetic shivering. In total, 16 studies including 1485 patients were analysed.

Ketamine was first synthesized in the early 1960s as a safe alternative to phencyclidine [26]. It is a non-competitive -NMDA receptor antagonist with an effect of thermoregulation. Other than being a competitive NMDA receptor antagonist, ketamine also acts as an opioid agonist [27]. Further, it can cause blockage of amine uptake in the descending inhibitory monoaminergic pain pathways, having a local anaesthetic action and interacting with the muscarinic receptors [28]. In contrast, even at sub-anaesthetic doses, ketamine might cause a dissociative state, characterised by a sense of detachment from one’s physical body and the external world (depersonalization and derealization). Ketamine probably controls shivering by acting on non-shivering thermogenesis [29]. Ketamine is predominantly utilized as an anaesthetic agent that induces analgesia but for a long time it has been criticized for some of its side effects which include the induction of a psychedelic state causing agitation and hallucinations [30].

The key findings of our analysis are as follows. Ketamine exposure was relatively better in reducing the occurrence of postanaesthetic shivering compared to placebo. Compared with tramadol and ondansetron, ketamine slightly lowered the incidence of postanaesthetic shivering although not significantly. The effect of ketamine on postanaesthetic shivering remained equally beneficial for both spinal and general types of anaesthesia. A dose of 0.5 mg/kg had an advance effect compared to 0.25 mg/kg on the postanaesthetic shivering rate. The effect remained constant for all types of surgical procedures including orthopaedic surgery, laparotomy, caesarean section, urological, ENT, and endoscopic surgeries. Compared to ketamine, pethidine showed a quicker responsive rate. However, sufficient data was not available in other studies to show any advantage of other pharmacological interventions.

Furthermore, we evaluated the side effects of the anaesthetic drugs and the role of ketamine in preventing or overcoming the effects. Moreover, the efficacy of ketamine was compared with a placebo. The side effects observed in the trials were nausea, vomiting, hypotension, bradycardia, and hallucinations. Ketamine showed a favourable outcome in reducing the incidence rate of hypotension and bradycardia as ketamine causes dose dependent direct stimulation of the CNS which leads to increased sympathetic nervous system stimulation followed by increased systemic blood pressure and heart rate.

However, there was no effect of ketamine in decreasing the incidence rate of nausea and vomiting as compared to placebo. As ketamine is known to have a hallucinogenic effect, it is considered to have a potential role in glutamatergic signalling in psychosis; therefore, the usage of ketamine is suggested to be associated with auditory and verbal hallucinations. In our comparative study, we also found that the rate of occurrence of hallucination episodes was much higher in patients receiving a ketamine dose of 0.5 mg/kg compared to a lower dose of 0.25 mg/kg. The hallucinogenic effect of ketamine was evident when compared with the placebo drugs, for which there was no incidence of hallucinations reported in any of the trials. Ketamine can cause sedation in postoperative patients and deep sedation is considered to be a severe adverse event. However, for those experiencing shivering, mild sedation may prevent them from hurting themselves. In our study, we paid special attention to the sedation score of patients, although pooled analysis was not conducted because of various sedation scales. We found that mild to moderate sedation was more commonly seen in patients receiving different doses of ketamine.

Besides various pharmacological interventions above, we noticed that active warming for elective caesarean delivery reduced the incidence of postoperative shivering and provided more stable perioperative temperature change ^[31]^. Accumulating evidence has shown that the active warming method including electric heating, water-circulating garments, forced-air, and radiant heating is effective in preventing post-anaesthetic shivering. The current American Society of Anesthesiologists Task Force on Postanesthetic Care guidelines recommend forced-air warming as a common method to reduce shivering in the perioperative setting ^[32]^. Future research should focus on combinations of pharmacological interventions with non-pharmacological methods to better solve this problem.

The major limitation of our study is that we could not study the hemodynamical changes related to ketamine usage as there were no standard criteria being followed by the trials causing irrelevancy and uneven data for comparing and evaluating precise outcomes in this regard. Second, the sample size of included trials was relatively small which may decrease generalisability of our conclusions. Third, the evidence level for our outcomes was low or very low. However, we believe that our study is of value because it provided clear evidence of the benefit of prophylactic ketamine intervention for preventing post-anaesthetic shivering which may be helpful in clinical practice.

## Conclusion

In this meta-analysis, we assessed various aspects of ketamine usage in controlling post-anaesthetic shivering. We found that ketamine reduced the incidence rate of shivering compared to the placebo. Although it is beneficial, it did not show any superiority over other pharmacological interventions. Ketamine is of clinical value, but further studies should be performed on a wider scale to determine more emphasized results. Furthermore, larger clinical trials investigating the combination of different anti-shivering regimens are warranted.

## Data Availability

The datasets used in the analysis was collected by online search, and the datasets analyzed in the current study are available from the corresponding author on reasonable request.

## Abbreviations

CNS: Central nervous system
ECG: Electrocardiography
ENT: Ear-nose-throat
NMDA: N-methy-D-aspartate
PONV: Postoperative nausea and vomiting
PRISMA: Reporting Items for Systematic Reviews and Meta-Analysis
SpO2: Oxygen saturation
